# (*E*)-1-(Thio­phen-2-yl)-3-(2,4,6-tri­meth­oxy­phen­yl)prop-2-en-1-one

**DOI:** 10.1107/S1600536811042930

**Published:** 2011-10-29

**Authors:** Hoong-Kun Fun, Thitipone Suwunwong, Teerasak Anantapong, Chatchanok Karalai, Suchada Chantrapromma

**Affiliations:** a X-ray Crystallography Unit, School of Physics, Universiti Sains Malaysia, 11800 USM, Penang, Malaysia; b Crystal Materials Research Unit, Department of Chemistry, Faculty of Science, Prince of Songkla University, Hat-Yai, Songkhla 90112, Thailand; c Department of Biotechnology, Faculty of Agro-Industry, Prince of Songkla University, Hat-Yai, Songkhla 90112, Thailand

## Abstract

There are two crystallograpically independent mol­ecules in the asymmetric unit of the title heteroaryl chalcone derivative, C_16_H_16_O_4_S, with slightly different conformations. The thienyl ring of one mol­ecule is disordered over two positions, with a refined site-occupancy ratio of 0.713 (5):0.287 (5). The mol­ecules are twisted: the dihedral angle between the thienyl and benzene rings is 9.72 (19)° in the ordered mol­ecule, and 3.8 (4) and 2.1 (8)° for the major and minor components, respectively, in the disordered mol­ecule. In both mol­ecules, all three substituted meth­oxy groups are coplanar with the benzene ring to which they are attached. In each mol­ecule, a weak intra­molecular C—H⋯O inter­action generates an *S*(6) ring motif. In the crystal structure, adjacent mol­ecules are linked into a three-dimensional network by weak C—H⋯O inter­actions.

## Related literature

For bond-length data, see: Allen *et al.* (1987 ▶). For related literature on hydrogen-bond motifs, see: Bernstein *et al.* (1995 ▶). For related structures, see: Chantrapromma *et al.* (2009 ▶); Fun *et al.* (2010 ▶, 2011 ▶); Suwunwong *et al.* (2009 ▶). For background to and applications of chalcones, see: Go *et al.* (2005 ▶); Liu *et al.* (2008 ▶); Ng *et al.* (2009 ▶); Ni *et al.* (2004 ▶); Suwunwong *et al.* (2011 ▶); Tewtrakul *et al.* (2003 ▶). For the stability of the temperature controller used in the data collection, see: Cosier & Glazer (1986 ▶).
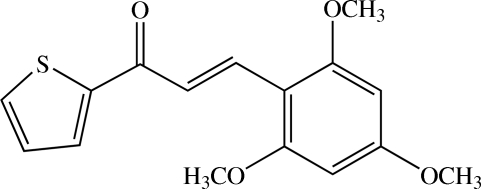


## Experimental

### 

#### Crystal data

C_16_H_16_O_4_S
*M* *_r_* = 304.36
Orthorhombic, 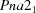

*a* = 22.8482 (10) Å
*b* = 31.2117 (13) Å
*c* = 3.9876 (2) Å
*V* = 2843.7 (2) Å^3^
*Z* = 8
Mo *K*α radiation
μ = 0.24 mm^−1^
*T* = 100 K
0.60 × 0.06 × 0.05 mm

#### Data collection

Bruker APEXII CCD area-detector diffractometer
Absorption correction: multi-scan (*SADABS*; Bruker, 2005 ▶) *T*_min_ = 0.869, *T*_max_ = 0.988
20029 measured reflections
8085 independent reflections
5348 reflections with *I* > 2σ(*I*)
*R*_int_ = 0.065

#### Refinement

*R*[*F* ^2^ > 2σ(*F* ^2^)] = 0.066
*wR*(*F* ^2^) = 0.178
*S* = 1.01
8085 reflections
402 parameters
1 restraint
H-atom parameters constrained
Δρ_max_ = 1.08 e Å^−3^
Δρ_min_ = −0.56 e Å^−3^
Absolute structure: Flack (1983 ▶), with 3389 Friedel pairs
Flack parameter: 0.09 (11)

### 

Data collection: *APEX2* (Bruker, 2005 ▶); cell refinement: *SAINT* (Bruker, 2005 ▶); data reduction: *SAINT*; program(s) used to solve structure: *SHELXTL* (Sheldrick, 2008 ▶); program(s) used to refine structure: *SHELXTL*; molecular graphics: *SHELXTL*; software used to prepare material for publication: *SHELXTL* and *PLATON* (Spek, 2009 ▶).

## Supplementary Material

Crystal structure: contains datablock(s) global, I. DOI: 10.1107/S1600536811042930/rz2650sup1.cif

Structure factors: contains datablock(s) I. DOI: 10.1107/S1600536811042930/rz2650Isup2.hkl

Supplementary material file. DOI: 10.1107/S1600536811042930/rz2650Isup3.cml

Additional supplementary materials:  crystallographic information; 3D view; checkCIF report

## Figures and Tables

**Table 1:** Hydrogen-bond geometry (Å, °)

*D*—H⋯*A*	*D*—H	H⋯*A*	*D*⋯*A*	*D*—H⋯*A*
C2*B*—H2*B*⋯O3*A*	0.93	2.56	3.482 (10)	171
C6*A*—H6*A*⋯O4*A*	0.93	2.22	2.815 (4)	121
C6*B*—H6*B*⋯O4*B*	0.93	2.24	2.824 (4)	120
C15*A*—H15*C*⋯O1*B*^i^	0.96	2.51	3.451 (5)	166
C15*B*—H15*F*⋯O1*A*^ii^	0.96	2.55	3.355 (5)	141
C16*B*—H16*E*⋯O3*B*^iii^	0.96	2.59	3.401 (4)	142

Symmetry codes: (i) 
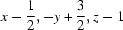
; (ii) 
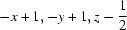
; (iii) 
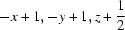
.

**Fig. 1.**
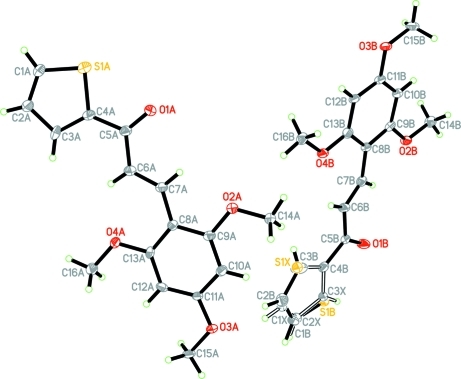
The molecular structure of the title compound, showing 50% probability displacement ellipsoids. Open bonds show the minor component of the disordered thienyl ring.

**Fig. 2.**
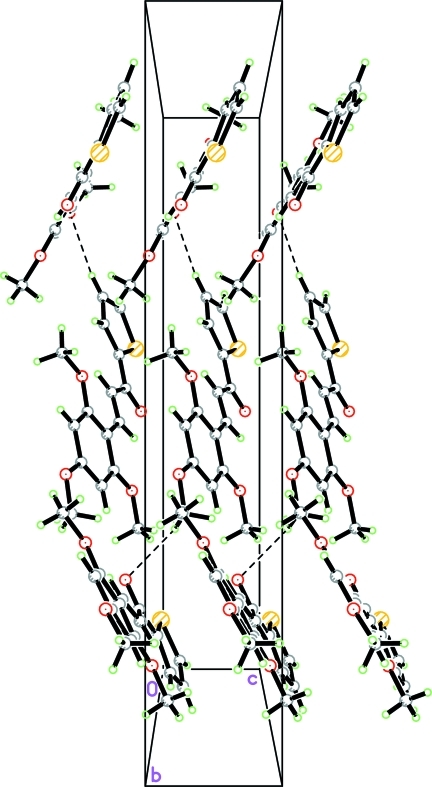
The crystal packing of the title compound viewed along the b axis. Only the major component of disorder is shown. Weak C—H···O interactions are shown as dashed lines.

## supplementary crystallographic information

### Comment

Chalcones have been reported to be responsible for a variety of biological activities such as analgesic, anti-inflammatory, antibacterial and antimycotic (Go *et al.*, 2005; Liu *et al.*, 2008; Ni *et al.*, 2004) as well as HIV-1 protease inhibitory (Tewtrakul *et al.*, 2003) and tyrosinase inhibitory (Ng *et al.*, 2009) properties. Our research on the fluorescent and biological studies of chalcones and heteroaryl chalcone derivatives (Chantrapromma *et al.*, 2009; Suwunwong *et al.*, 2009, 2011) led us to synthesize the title heteroaryl chalcone (I). (I) exhibits fluorescent property (Suwunwong *et al.*, 2011) and possess moderate analgesic property. It was also tested for antibacterial activities but found to be inactive. Herein we report the crystal structure of (I).

There are two crystallographic independent molecules *A* and *B* in the asymmetric unit of (I) with different conformations of the methoxy group at *para* position or at atom C11 and also in bond angles (Fig. 1). The thienyl ring of molecule *B* is disordered over two orientations with the refined site-occupancy ratio of 0.713 (5):0.287 (5). The thienyl rings in the major and minor components are related by 180° rotation. The molecule of (I) is slightly twisted. The dihedral angle between the thienyl and benzene rings is 9.72 (19)° in molecule *A* whereas these values are 3.8 (4) and 2.1 (8)° for the major and minor components in the disordered molecule *B*. The central prop-2-en-1-one bridge (C5–C7/O1) in both molecules is slightly twisted as indicated by the torsion angle O1—C5—C6—C7 = 5.8 (6) and 6.6 (6)° in molecules *A* and *B*, respectively. The mean plane through this bridge makes dihedral angles of 8.9 (3) and 2.3 (2)° with the thienyl and benzene rings, respectively, in molecule *A* whereas the corresponding values are 4.2 (4) and 8.0 (3)° in molecule *B* for the major component, and 8.2 (8) and 8.0 (3)° for the minor component. In both molecules, all the three substituted methoxy groups are co-planar with the attached benzene with torsion angles C14—O2—C9—C10 = -5.3 (5)°; C15—O3—C11—C12 = 3.7 (5)° and C16—O4—C13—C12 = 0.3 (5)° in molecule *A*. The corresponding values are 0.6 (5), 178.0 (3) and 0.6 (5)° in molecule *B*. This also indicates that the methoxy group at the *para* position (or at atom C11) has different conformations as it points toward the methoxy group at the *ortho* position at atom C13 (in molecule *A*) whereas it points toward the *ortho* methoxy at atom C9 (in molecule *B*). In each molecule, intramolecular C—H···O weak interaction (Table 1) generates S(6) ring motif (Bernstein *et al.*, 1995). The bond distances agree with the literature values (Allen *et al.*, 1987) and are comparable with those observed in related structures (Chantrapromma *et al.*, 2009; Fun *et al.*, 2010, 2011; Suwunwong *et al.*, 2009).

In the crystal packing (Fig. 2), adjacent molecules are linked into a three-dimensional network by weak C—H···O interactions (Table 1).

### Experimental

The title compound was synthesized by the condensation of 2,4,6-trimethoxybenzaldehyde (0.40 g, 2 mmol) with 2-acethylthiophene (0.35 ml, 2 mmol) in ethanol (30 ml) in the presence of 30% NaOH (aq) (5 ml). After stirring for 3 h in ice bath at 278 K, the resulting pale-yellow solid was collected by filtration, washed with distilled water, dried in air and purified by recrystallization from acetone. Pale-yellow needle-shaped single crystals of the title compound suitable for X-ray structure determination were recrystallized from acetone–ethanol (1:1 *v*/*v*) by slow evaporation of the solvent at room temperature after several days; m.p. 381–382 K.

### Refinement

All H atoms were placed in calculated positions, with C—H = 0.93 Å, *U*_iso_ = 1.2*U*_eq_(C) for aromatic and methyne C atoms and C—H = 0.96 Å, *U*_iso_ = 1.5*U*_eq_(C) for methyl groups. A rotating group model was used for the methyl groups. The highest residual electron density peak is located at 1.65 Å from C3 and the deepest hole is located at 0.29 Å from S1A. The thienyl ring of molecule B is disordered over two sites with refined site occupancies of 0.713 (5) and 0.287 (5). Initially SAME, DELU and SIMU restraints were used. In the final refinement, these restraints were removed. A total of 3389 Friedel pairs were used to determine the absolute structure.

### Crystal data

**Table:**

C_16_H_16_O_4_S	*D*_x_ = 1.422 Mg m^−^^3^
*M**_r_* = 304.36	Melting point = 381–382 K
Orthorhombic, *P**n**a*2_1_	Mo *K*α radiation, λ = 0.71073 Å
Hall symbol: P 2c -2n	Cell parameters from 8085 reflections
*a* = 22.8482 (10) Å	θ = 1.3–30.0°
*b* = 31.2117 (13) Å	µ = 0.24 mm^−^^1^
*c* = 3.9876 (2) Å	*T* = 100 K
*V* = 2843.7 (2) Å^3^	Needle, pale yellow
*Z* = 8	0.60 × 0.06 × 0.05 mm
*F*(000) = 1280	

### Data collection

**Table:**

Bruker APEXII CCD area-detector diffractometer	8085 independent reflections
Radiation source: sealed tube	5348 reflections with *I* > 2σ(*I*)
graphite	*R*_int_ = 0.065
φ and ω scans	θ_max_ = 30.0°, θ_min_ = 1.3°
Absorption correction: multi-scan (*SADABS*; Bruker, 2005)	*h* = −27→32
*T*_min_ = 0.869, *T*_max_ = 0.988	*k* = −43→40
20029 measured reflections	*l* = −5→5

### Refinement

**Table:**

Refinement on *F*^2^	Secondary atom site location: difference Fourier map
Least-squares matrix: full	Hydrogen site location: inferred from neighbouring sites
*R*[*F*^2^ > 2σ(*F*^2^)] = 0.066	H-atom parameters constrained
*wR*(*F*^2^) = 0.178	*w* = 1/[σ^2^(*F*_o_^2^) + (0.0951*P*)^2^] where *P* = (*F*_o_^2^ + 2*F*_c_^2^)/3
*S* = 1.01	(Δ/σ)_max_ = 0.001
8085 reflections	Δρ_max_ = 1.08 e Å^−^^3^
402 parameters	Δρ_min_ = −0.56 e Å^−^^3^
1 restraint	Absolute structure: Flack (1983), with 3389 Friedel pairs
Primary atom site location: structure-invariant direct methods	Flack parameter: 0.09 (11)

### Special details

**Table:**

Experimental. The crystal was placed in the cold stream of an Oxford Cryosystems Cobra open-flow nitrogen cryostat (Cosier & Glazer, 1986) operating at 120.0 (1) K.
Geometry. All esds (except the esd in the dihedral angle between two l.s. planes) are estimated using the full covariance matrix. The cell esds are taken into account individually in the estimation of esds in distances, angles and torsion angles; correlations between esds in cell parameters are only used when they are defined by crystal symmetry. An approximate (isotropic) treatment of cell esds is used for estimating esds involving l.s. planes.
Refinement. Refinement of F^2^ against ALL reflections. The weighted R-factor wR and goodness of fit S are based on F^2^, conventional R-factors R are based on F, with F set to zero for negative F^2^. The threshold expression of F^2^ > 2sigma(F^2^) is used only for calculating R-factors(gt) etc. and is not relevant to the choice of reflections for refinement. R-factors based on F^2^ are statistically about twice as large as those based on F, and R- factors based on ALL data will be even larger.

### Fractional atomic coordinates and isotropic or equivalent isotropic displacement parameters (Å^2^)

**Table:**

	*x*	*y*	*z*	*U*_iso_*/*U*_eq_	Occ. (<1)
S1A	0.13970 (4)	0.41398 (3)	1.0329 (3)	0.0270 (2)	
O1A	0.21057 (10)	0.48128 (7)	0.7262 (8)	0.0228 (6)	
O2A	0.27887 (11)	0.61480 (7)	0.4202 (7)	0.0213 (6)	
O3A	0.19621 (10)	0.75129 (7)	0.6341 (7)	0.0206 (6)	
O4A	0.09780 (10)	0.62191 (7)	0.9862 (7)	0.0211 (6)	
C1A	0.07345 (15)	0.40255 (11)	1.2033 (10)	0.0217 (8)	
H1A	0.0622	0.3752	1.2684	0.026*	
C2A	0.03841 (16)	0.43786 (10)	1.2349 (11)	0.0241 (8)	
H2A	0.0005	0.4367	1.3198	0.029*	
C3A	0.06544 (15)	0.47698 (12)	1.1249 (10)	0.0236 (8)	
H3A	0.0483	0.5040	1.1312	0.028*	
C4A	0.12232 (15)	0.46779 (10)	1.0057 (10)	0.0192 (7)	
C5A	0.16654 (15)	0.49669 (10)	0.8497 (10)	0.0198 (7)	
C6A	0.15415 (15)	0.54312 (10)	0.8687 (10)	0.0209 (8)	
H6A	0.1214	0.5529	0.9841	0.025*	
C7A	0.19013 (15)	0.57103 (10)	0.7198 (10)	0.0187 (7)	
H7A	0.2213	0.5587	0.6051	0.022*	
C8A	0.18848 (15)	0.61784 (10)	0.7077 (10)	0.0166 (7)	
C9A	0.23464 (15)	0.64009 (10)	0.5441 (10)	0.0175 (7)	
C10A	0.23602 (15)	0.68428 (10)	0.5233 (10)	0.0177 (7)	
H10A	0.2669	0.6980	0.4162	0.021*	
C11A	0.19052 (15)	0.70813 (10)	0.6647 (10)	0.0170 (7)	
C12A	0.14394 (15)	0.68798 (10)	0.8199 (10)	0.0190 (7)	
H12A	0.1135	0.7040	0.9108	0.023*	
C13A	0.14324 (14)	0.64351 (10)	0.8385 (10)	0.0185 (7)	
C14A	0.32476 (14)	0.63633 (10)	0.2398 (10)	0.0190 (7)	
H14A	0.3511	0.6155	0.1470	0.029*	
H14B	0.3458	0.6548	0.3900	0.029*	
H14C	0.3080	0.6531	0.0619	0.029*	
C15A	0.15164 (15)	0.77720 (10)	0.7938 (11)	0.0208 (8)	
H15A	0.1602	0.8070	0.7584	0.031*	
H15B	0.1511	0.7713	1.0300	0.031*	
H15C	0.1141	0.7705	0.6989	0.031*	
C16A	0.05059 (16)	0.64661 (11)	1.1205 (11)	0.0232 (8)	
H16A	0.0221	0.6278	1.2182	0.035*	
H16B	0.0327	0.6629	0.9441	0.035*	
H16C	0.0653	0.6657	1.2892	0.035*	
S1B	0.42377 (10)	0.79935 (8)	1.2577 (7)	0.0198 (5)	0.713 (5)
O1B	0.52996 (11)	0.74766 (7)	1.3193 (8)	0.0283 (7)	
O2B	0.65708 (9)	0.63419 (7)	1.2581 (7)	0.0199 (6)	
O3B	0.62447 (10)	0.49871 (7)	0.7051 (7)	0.0192 (5)	
O4B	0.47249 (10)	0.60034 (7)	0.8092 (7)	0.0194 (5)	
C1B	0.3535 (4)	0.8012 (3)	1.105 (2)	0.0191 (18)	0.713 (5)
H1B	0.3290	0.8248	1.1252	0.023*	0.713 (5)
C2B	0.3376 (4)	0.7629 (3)	0.947 (3)	0.027 (2)	0.713 (5)
H2B	0.3018	0.7578	0.8433	0.032*	0.713 (5)
C3B	0.3838 (5)	0.7331 (4)	0.968 (3)	0.032 (3)	0.713 (5)
H3B	0.3804	0.7050	0.8923	0.038*	0.713 (5)
S1X	0.3779 (3)	0.7288 (2)	0.9029 (17)	0.0168 (13)*	0.287 (5)
C1X	0.3356 (11)	0.7722 (7)	0.978 (7)	0.013 (5)*	0.287 (5)
H1BX	0.2977	0.7753	0.8964	0.015*	0.287 (5)
C2X	0.3631 (11)	0.8023 (9)	1.170 (6)	0.018 (6)*	0.287 (5)
H2BX	0.3451	0.8268	1.2552	0.022*	0.287 (5)
C3X	0.4244 (12)	0.7909 (7)	1.225 (7)	0.014 (6)*	0.287 (5)
H3BX	0.4524	0.8086	1.3225	0.017*	0.287 (5)
C4B	0.43447 (15)	0.74896 (10)	1.1089 (10)	0.0189 (7)	
C5B	0.49125 (15)	0.72746 (10)	1.1768 (10)	0.0195 (8)	
C6B	0.49764 (15)	0.68308 (10)	1.0567 (11)	0.0213 (8)	
H6B	0.4682	0.6704	0.9301	0.026*	
C7B	0.54678 (15)	0.66094 (10)	1.1323 (10)	0.0200 (8)	
H7B	0.5731	0.6754	1.2698	0.024*	
C8B	0.56499 (15)	0.61804 (9)	1.0324 (10)	0.0175 (7)	
C9B	0.62300 (16)	0.60488 (10)	1.0981 (10)	0.0188 (7)	
C10B	0.64515 (15)	0.56527 (10)	0.9991 (11)	0.0192 (7)	
H10B	0.6836	0.5575	1.0465	0.023*	
C11B	0.60766 (15)	0.53768 (10)	0.8260 (10)	0.0182 (7)	
C12B	0.54957 (14)	0.54812 (10)	0.7577 (10)	0.0176 (7)	
H12B	0.5253	0.5291	0.6446	0.021*	
C13B	0.52869 (15)	0.58828 (11)	0.8645 (10)	0.0184 (7)	
C14B	0.71693 (15)	0.62351 (11)	1.3201 (11)	0.0239 (8)	
H14D	0.7362	0.6474	1.4246	0.036*	
H14E	0.7360	0.6169	1.1117	0.036*	
H14F	0.7188	0.5991	1.4657	0.036*	
C15B	0.68437 (15)	0.48626 (11)	0.7563 (12)	0.0270 (9)	
H15D	0.6916	0.4596	0.6443	0.040*	
H15E	0.6917	0.4830	0.9920	0.040*	
H15F	0.7098	0.5079	0.6669	0.040*	
C16B	0.43513 (15)	0.57035 (11)	0.6405 (10)	0.0209 (8)	
H16D	0.3961	0.5817	0.6297	0.031*	
H16E	0.4348	0.5438	0.7615	0.031*	
H16F	0.4495	0.5655	0.4174	0.031*	

### Atomic displacement parameters (Å^2^)

**Table:**

	*U*^11^	*U*^22^	*U*^33^	*U*^12^	*U*^13^	*U*^23^
S1A	0.0279 (5)	0.0170 (4)	0.0359 (6)	−0.0005 (4)	0.0025 (5)	0.0010 (4)
O1A	0.0172 (11)	0.0159 (11)	0.0354 (17)	0.0015 (9)	0.0028 (12)	−0.0011 (11)
O2A	0.0188 (12)	0.0160 (11)	0.0290 (15)	0.0019 (9)	0.0044 (11)	0.0041 (10)
O3A	0.0194 (12)	0.0099 (10)	0.0326 (16)	0.0006 (9)	0.0018 (11)	0.0029 (10)
O4A	0.0174 (12)	0.0135 (11)	0.0324 (16)	−0.0022 (9)	0.0064 (12)	−0.0005 (11)
C1A	0.0233 (17)	0.0186 (16)	0.023 (2)	−0.0046 (14)	0.0012 (16)	0.0030 (14)
C2A	0.0229 (17)	0.0171 (16)	0.032 (2)	−0.0014 (13)	0.0067 (18)	0.0011 (16)
C3A	0.0213 (18)	0.0237 (17)	0.026 (2)	−0.0096 (14)	0.0046 (16)	0.0041 (15)
C4A	0.0231 (17)	0.0125 (14)	0.0219 (19)	−0.0005 (12)	−0.0008 (16)	−0.0015 (14)
C5A	0.0195 (17)	0.0135 (15)	0.026 (2)	0.0001 (12)	−0.0029 (16)	−0.0021 (14)
C6A	0.0208 (17)	0.0131 (15)	0.029 (2)	0.0009 (12)	0.0005 (16)	−0.0023 (14)
C7A	0.0206 (16)	0.0141 (14)	0.0214 (19)	0.0022 (12)	0.0010 (15)	0.0019 (15)
C8A	0.0159 (15)	0.0132 (14)	0.0207 (19)	−0.0015 (12)	−0.0011 (14)	−0.0012 (13)
C9A	0.0201 (16)	0.0141 (14)	0.0181 (18)	0.0018 (12)	−0.0025 (15)	0.0027 (14)
C10A	0.0186 (16)	0.0145 (14)	0.0199 (18)	−0.0024 (12)	−0.0009 (15)	0.0019 (14)
C11A	0.0148 (15)	0.0098 (14)	0.026 (2)	0.0010 (12)	−0.0032 (14)	0.0013 (13)
C12A	0.0163 (15)	0.0144 (15)	0.026 (2)	0.0043 (12)	−0.0027 (15)	0.0001 (14)
C13A	0.0123 (15)	0.0177 (15)	0.026 (2)	−0.0050 (12)	−0.0013 (14)	0.0003 (14)
C14A	0.0170 (16)	0.0170 (15)	0.023 (2)	0.0005 (12)	0.0005 (16)	0.0023 (15)
C15A	0.0229 (17)	0.0099 (14)	0.030 (2)	0.0017 (12)	−0.0045 (16)	0.0019 (15)
C16A	0.0217 (18)	0.0180 (16)	0.030 (2)	−0.0017 (13)	0.0027 (16)	−0.0041 (15)
S1B	0.0189 (8)	0.0084 (8)	0.0322 (10)	0.0019 (7)	0.0018 (7)	−0.0026 (8)
O1B	0.0207 (12)	0.0134 (11)	0.051 (2)	0.0005 (9)	−0.0075 (14)	−0.0025 (12)
O2B	0.0140 (11)	0.0120 (10)	0.0339 (16)	−0.0022 (8)	−0.0061 (12)	−0.0020 (11)
O3B	0.0193 (12)	0.0106 (10)	0.0276 (15)	0.0017 (8)	−0.0013 (11)	0.0009 (10)
O4B	0.0166 (11)	0.0112 (10)	0.0304 (16)	−0.0005 (8)	−0.0018 (11)	−0.0001 (10)
C1B	0.016 (4)	0.020 (3)	0.022 (5)	0.005 (2)	−0.004 (3)	0.005 (3)
C2B	0.027 (4)	0.020 (4)	0.033 (5)	−0.004 (3)	0.003 (3)	−0.008 (4)
C3B	0.044 (5)	0.021 (4)	0.031 (6)	0.004 (3)	−0.007 (4)	0.001 (4)
C4B	0.0195 (17)	0.0149 (15)	0.022 (2)	−0.0038 (13)	0.0001 (15)	0.0014 (14)
C5B	0.0176 (16)	0.0120 (15)	0.029 (2)	−0.0003 (13)	0.0051 (15)	0.0019 (13)
C6B	0.0210 (17)	0.0118 (14)	0.031 (2)	−0.0005 (12)	−0.0013 (17)	−0.0014 (16)
C7B	0.0179 (16)	0.0152 (15)	0.027 (2)	−0.0043 (13)	−0.0013 (15)	−0.0005 (14)
C8B	0.0196 (16)	0.0113 (13)	0.0215 (18)	−0.0004 (12)	0.0031 (15)	−0.0009 (14)
C9B	0.0216 (17)	0.0143 (15)	0.020 (2)	−0.0038 (13)	0.0010 (15)	0.0039 (13)
C10B	0.0184 (16)	0.0136 (15)	0.026 (2)	−0.0001 (12)	0.0005 (16)	0.0022 (15)
C11B	0.0218 (17)	0.0103 (14)	0.022 (2)	−0.0003 (12)	0.0023 (15)	0.0003 (13)
C12B	0.0196 (15)	0.0103 (13)	0.0229 (19)	−0.0015 (12)	−0.0013 (16)	0.0023 (14)
C13B	0.0198 (17)	0.0169 (15)	0.0184 (18)	−0.0013 (13)	0.0021 (15)	−0.0001 (14)
C14B	0.0178 (16)	0.0197 (16)	0.034 (2)	−0.0041 (13)	−0.0060 (17)	0.0000 (16)
C15B	0.0227 (18)	0.0125 (15)	0.046 (3)	0.0051 (13)	−0.0004 (19)	0.0013 (18)
C16B	0.0190 (17)	0.0185 (16)	0.025 (2)	−0.0040 (13)	−0.0026 (15)	0.0000 (14)

### Geometric parameters (Å, °)

**Table:**

S1A—C1A	1.697 (4)	O2B—C14B	1.429 (4)
S1A—C4A	1.729 (3)	O3B—C11B	1.364 (4)
O1A—C5A	1.219 (4)	O3B—C15B	1.437 (4)
O2A—C9A	1.374 (4)	O4B—C13B	1.356 (4)
O2A—C14A	1.438 (4)	O4B—C16B	1.434 (4)
O3A—C11A	1.359 (4)	C1B—C2B	1.399 (13)
O3A—C15A	1.448 (4)	C1B—H1B	0.9300
O4A—C13A	1.371 (4)	C2B—C3B	1.408 (15)
O4A—C16A	1.430 (4)	C2B—H2B	0.9300
C1A—C2A	1.368 (5)	C3B—C4B	1.380 (12)
C1A—H1A	0.9300	C3B—H3B	0.9300
C2A—C3A	1.437 (5)	S1X—C4B	1.657 (7)
C2A—H2A	0.9300	S1X—C1X	1.69 (2)
C3A—C4A	1.413 (5)	C1X—C2X	1.37 (3)
C3A—H3A	0.9300	C1X—H1BX	0.9300
C4A—C5A	1.491 (5)	C2X—C3X	1.46 (3)
C5A—C6A	1.478 (4)	C2X—H2BX	0.9300
C6A—C7A	1.337 (5)	C3X—C4B	1.41 (2)
C6A—H6A	0.9300	C3X—H3BX	0.9300
C7A—C8A	1.462 (4)	C4B—C5B	1.485 (5)
C7A—H7A	0.9300	C5B—C6B	1.473 (5)
C8A—C13A	1.408 (5)	C6B—C7B	1.353 (5)
C8A—C9A	1.421 (5)	C6B—H6B	0.9300
C9A—C10A	1.382 (4)	C7B—C8B	1.458 (4)
C10A—C11A	1.397 (5)	C7B—H7B	0.9300
C10A—H10A	0.9300	C8B—C9B	1.412 (5)
C11A—C12A	1.383 (5)	C8B—C13B	1.414 (5)
C12A—C13A	1.390 (4)	C9B—C10B	1.393 (5)
C12A—H12A	0.9300	C10B—C11B	1.397 (5)
C14A—H14A	0.9600	C10B—H10B	0.9300
C14A—H14B	0.9600	C11B—C12B	1.394 (5)
C14A—H14C	0.9600	C12B—C13B	1.407 (5)
C15A—H15A	0.9600	C12B—H12B	0.9300
C15A—H15B	0.9600	C14B—H14D	0.9600
C15A—H15C	0.9600	C14B—H14E	0.9600
C16A—H16A	0.9600	C14B—H14F	0.9600
C16A—H16B	0.9600	C15B—H15D	0.9600
C16A—H16C	0.9600	C15B—H15E	0.9600
S1B—C4B	1.699 (4)	C15B—H15F	0.9600
S1B—C1B	1.717 (10)	C16B—H16D	0.9600
O1B—C5B	1.226 (4)	C16B—H16E	0.9600
O2B—C9B	1.360 (4)	C16B—H16F	0.9600
			
C1A—S1A—C4A	91.40 (17)	C1B—C2B—H2B	125.0
C9A—O2A—C14A	116.6 (3)	C3B—C2B—H2B	125.0
C11A—O3A—C15A	116.5 (3)	C4B—C3B—C2B	114.6 (9)
C13A—O4A—C16A	117.8 (3)	C4B—C3B—H3B	122.7
C2A—C1A—S1A	112.9 (3)	C2B—C3B—H3B	122.7
C2A—C1A—H1A	123.5	C4B—S1X—C1X	93.0 (9)
S1A—C1A—H1A	123.5	C2X—C1X—S1X	113 (2)
C1A—C2A—C3A	113.9 (3)	C2X—C1X—H1BX	123.6
C1A—C2A—H2A	123.1	S1X—C1X—H1BX	123.6
C3A—C2A—H2A	123.1	C1X—C2X—C3X	111 (2)
C4A—C3A—C2A	109.0 (3)	C1X—C2X—H2BX	124.6
C4A—C3A—H3A	125.5	C3X—C2X—H2BX	124.6
C2A—C3A—H3A	125.5	C4B—C3X—C2X	110 (2)
C3A—C4A—C5A	129.9 (3)	C4B—C3X—H3BX	125.2
C3A—C4A—S1A	112.8 (3)	C2X—C3X—H3BX	125.2
C5A—C4A—S1A	117.3 (3)	C3B—C4B—C3X	109.3 (12)
O1A—C5A—C6A	124.4 (3)	C3B—C4B—C5B	130.2 (6)
O1A—C5A—C4A	119.3 (3)	C3X—C4B—C5B	120.2 (12)
C6A—C5A—C4A	116.2 (3)	C3X—C4B—S1X	112.9 (12)
C7A—C6A—C5A	119.9 (3)	C5B—C4B—S1X	126.9 (4)
C7A—C6A—H6A	120.1	C3B—C4B—S1B	110.7 (5)
C5A—C6A—H6A	120.1	C5B—C4B—S1B	118.7 (3)
C6A—C7A—C8A	130.5 (3)	S1X—C4B—S1B	114.4 (3)
C6A—C7A—H7A	114.7	O1B—C5B—C6B	124.3 (3)
C8A—C7A—H7A	114.7	O1B—C5B—C4B	118.8 (3)
C13A—C8A—C9A	115.9 (3)	C6B—C5B—C4B	116.9 (3)
C13A—C8A—C7A	125.1 (3)	C7B—C6B—C5B	119.4 (3)
C9A—C8A—C7A	119.0 (3)	C7B—C6B—H6B	120.3
O2A—C9A—C10A	122.3 (3)	C5B—C6B—H6B	120.3
O2A—C9A—C8A	115.5 (3)	C6B—C7B—C8B	130.2 (3)
C10A—C9A—C8A	122.1 (3)	C6B—C7B—H7B	114.9
C9A—C10A—C11A	119.4 (3)	C8B—C7B—H7B	114.9
C9A—C10A—H10A	120.3	C9B—C8B—C13B	116.6 (3)
C11A—C10A—H10A	120.3	C9B—C8B—C7B	119.0 (3)
O3A—C11A—C12A	124.4 (3)	C13B—C8B—C7B	124.4 (3)
O3A—C11A—C10A	114.9 (3)	O2B—C9B—C10B	121.5 (3)
C12A—C11A—C10A	120.7 (3)	O2B—C9B—C8B	115.4 (3)
C11A—C12A—C13A	119.1 (3)	C10B—C9B—C8B	123.1 (3)
C11A—C12A—H12A	120.4	C9B—C10B—C11B	117.7 (3)
C13A—C12A—H12A	120.4	C9B—C10B—H10B	121.2
O4A—C13A—C12A	121.5 (3)	C11B—C10B—H10B	121.2
O4A—C13A—C8A	115.8 (3)	O3B—C11B—C12B	114.1 (3)
C12A—C13A—C8A	122.7 (3)	O3B—C11B—C10B	123.5 (3)
O2A—C14A—H14A	109.5	C12B—C11B—C10B	122.4 (3)
O2A—C14A—H14B	109.5	C11B—C12B—C13B	118.2 (3)
H14A—C14A—H14B	109.5	C11B—C12B—H12B	120.9
O2A—C14A—H14C	109.5	C13B—C12B—H12B	120.9
H14A—C14A—H14C	109.5	O4B—C13B—C12B	121.3 (3)
H14B—C14A—H14C	109.5	O4B—C13B—C8B	116.7 (3)
O3A—C15A—H15A	109.5	C12B—C13B—C8B	122.0 (3)
O3A—C15A—H15B	109.5	O2B—C14B—H14D	109.5
H15A—C15A—H15B	109.5	O2B—C14B—H14E	109.5
O3A—C15A—H15C	109.5	H14D—C14B—H14E	109.5
H15A—C15A—H15C	109.5	O2B—C14B—H14F	109.5
H15B—C15A—H15C	109.5	H14D—C14B—H14F	109.5
O4A—C16A—H16A	109.5	H14E—C14B—H14F	109.5
O4A—C16A—H16B	109.5	O3B—C15B—H15D	109.5
H16A—C16A—H16B	109.5	O3B—C15B—H15E	109.5
O4A—C16A—H16C	109.5	H15D—C15B—H15E	109.5
H16A—C16A—H16C	109.5	O3B—C15B—H15F	109.5
H16B—C16A—H16C	109.5	H15D—C15B—H15F	109.5
C4B—S1B—C1B	92.4 (4)	H15E—C15B—H15F	109.5
C9B—O2B—C14B	118.2 (3)	O4B—C16B—H16D	109.5
C11B—O3B—C15B	117.3 (3)	O4B—C16B—H16E	109.5
C13B—O4B—C16B	117.3 (3)	H16D—C16B—H16E	109.5
C2B—C1B—S1B	112.0 (7)	O4B—C16B—H16F	109.5
C2B—C1B—H1B	124.0	H16D—C16B—H16F	109.5
S1B—C1B—H1B	124.0	H16E—C16B—H16F	109.5
C1B—C2B—C3B	110.0 (10)		
			
C4A—S1A—C1A—C2A	1.0 (3)	C2B—C3B—C4B—S1B	6.2 (11)
S1A—C1A—C2A—C3A	−1.3 (5)	C2X—C3X—C4B—C3B	3(2)
C1A—C2A—C3A—C4A	0.9 (5)	C2X—C3X—C4B—C5B	−171.8 (15)
C2A—C3A—C4A—C5A	176.5 (4)	C2X—C3X—C4B—S1X	8(2)
C2A—C3A—C4A—S1A	−0.2 (5)	C1X—S1X—C4B—C3B	51 (7)
C1A—S1A—C4A—C3A	−0.5 (3)	C1X—S1X—C4B—C3X	−3.7 (16)
C1A—S1A—C4A—C5A	−177.6 (3)	C1X—S1X—C4B—C5B	176.0 (10)
C3A—C4A—C5A—O1A	−171.1 (4)	C1X—S1X—C4B—S1B	−3.0 (10)
S1A—C4A—C5A—O1A	5.4 (5)	C1B—S1B—C4B—C3B	−4.2 (7)
C3A—C4A—C5A—C6A	11.1 (6)	C1B—S1B—C4B—C5B	−178.0 (4)
S1A—C4A—C5A—C6A	−172.3 (3)	C1B—S1B—C4B—S1X	1.1 (5)
O1A—C5A—C6A—C7A	5.8 (6)	C3B—C4B—C5B—O1B	−176.9 (7)
C4A—C5A—C6A—C7A	−176.6 (4)	C3X—C4B—C5B—O1B	−3.9 (14)
C5A—C6A—C7A—C8A	−178.5 (4)	S1X—C4B—C5B—O1B	176.4 (5)
C6A—C7A—C8A—C13A	−5.4 (7)	S1B—C4B—C5B—O1B	−4.6 (5)
C6A—C7A—C8A—C9A	176.6 (4)	C3B—C4B—C5B—C6B	4.9 (9)
C14A—O2A—C9A—C10A	−5.3 (5)	C3X—C4B—C5B—C6B	177.9 (13)
C14A—O2A—C9A—C8A	177.2 (3)	S1X—C4B—C5B—C6B	−1.7 (6)
C13A—C8A—C9A—O2A	179.4 (3)	S1B—C4B—C5B—C6B	177.3 (3)
C7A—C8A—C9A—O2A	−2.4 (5)	O1B—C5B—C6B—C7B	6.6 (6)
C13A—C8A—C9A—C10A	1.9 (5)	C4B—C5B—C6B—C7B	−175.3 (4)
C7A—C8A—C9A—C10A	−179.9 (4)	C5B—C6B—C7B—C8B	−176.7 (4)
O2A—C9A—C10A—C11A	−177.8 (4)	C6B—C7B—C8B—C9B	169.3 (4)
C8A—C9A—C10A—C11A	−0.5 (6)	C6B—C7B—C8B—C13B	−9.2 (7)
C15A—O3A—C11A—C12A	3.7 (5)	C14B—O2B—C9B—C10B	0.6 (5)
C15A—O3A—C11A—C10A	−176.6 (3)	C14B—O2B—C9B—C8B	−177.6 (3)
C9A—C10A—C11A—O3A	179.4 (3)	C13B—C8B—C9B—O2B	179.6 (3)
C9A—C10A—C11A—C12A	−1.0 (6)	C7B—C8B—C9B—O2B	1.0 (5)
O3A—C11A—C12A—C13A	−179.4 (3)	C13B—C8B—C9B—C10B	1.5 (6)
C10A—C11A—C12A—C13A	1.0 (6)	C7B—C8B—C9B—C10B	−177.1 (4)
C16A—O4A—C13A—C12A	0.3 (5)	O2B—C9B—C10B—C11B	−177.9 (3)
C16A—O4A—C13A—C8A	−179.5 (3)	C8B—C9B—C10B—C11B	0.2 (6)
C11A—C12A—C13A—O4A	−179.2 (4)	C15B—O3B—C11B—C12B	178.0 (3)
C11A—C12A—C13A—C8A	0.6 (6)	C15B—O3B—C11B—C10B	−1.0 (5)
C9A—C8A—C13A—O4A	177.9 (3)	C9B—C10B—C11B—O3B	177.6 (3)
C7A—C8A—C13A—O4A	−0.2 (6)	C9B—C10B—C11B—C12B	−1.4 (6)
C9A—C8A—C13A—C12A	−1.9 (5)	O3B—C11B—C12B—C13B	−178.2 (3)
C7A—C8A—C13A—C12A	−180.0 (4)	C10B—C11B—C12B—C13B	0.9 (6)
C4B—S1B—C1B—C2B	1.4 (8)	C16B—O4B—C13B—C12B	0.6 (5)
S1B—C1B—C2B—C3B	1.8 (12)	C16B—O4B—C13B—C8B	−179.7 (3)
C1B—C2B—C3B—C4B	−5.1 (14)	C11B—C12B—C13B—O4B	−179.4 (3)
C4B—S1X—C1X—C2X	−2(2)	C11B—C12B—C13B—C8B	0.9 (6)
S1X—C1X—C2X—C3X	7(3)	C9B—C8B—C13B—O4B	178.2 (3)
C1X—C2X—C3X—C4B	−9(3)	C7B—C8B—C13B—O4B	−3.2 (6)
C2B—C3B—C4B—C3X	5.4 (16)	C9B—C8B—C13B—C12B	−2.1 (6)
C2B—C3B—C4B—C5B	179.0 (7)	C7B—C8B—C13B—C12B	176.5 (4)
C2B—C3B—C4B—S1X	−121 (7)		

### Hydrogen-bond geometry (Å, °)

**Table:**

*D*—H···*A*	*D*—H	H···*A*	*D*···*A*	*D*—H···*A*
C2B—H2B···O3A	0.93	2.56	3.482 (10)	171
C6A—H6A···O4A	0.93	2.22	2.815 (4)	121
C6B—H6B···O4B	0.93	2.24	2.824 (4)	120
C15A—H15C···O1B^i^	0.96	2.51	3.451 (5)	166
C15B—H15F···O1A^ii^	0.96	2.55	3.355 (5)	141
C16B—H16E···O3B^iii^	0.96	2.59	3.401 (4)	142

Symmetry codes:  (i) *x*−1/2, −*y*+3/2, *z*−1; (ii) −*x*+1, −*y*+1, *z*−1/2; (iii) −*x*+1, −*y*+1, *z*+1/2.
